# Age and Breed Associated Differences in the Duodenal Microbiota of European Meat Pigeons and Shiqi Pigeons and Their Associations with Growth Traits

**DOI:** 10.3390/ani16193024

**Published:** 2026-09-25

**Authors:** Liheng Zhang, Leshan Guo, Binglong Lu, Ruiting Li, Yang Niu, Qianqian Li, Yiming Zhang, Mengru Wu, Bin Wei, Pengkun Yang, Xinlei Wang, Liyu Yang, Yanzhang Gong

**Affiliations:** 1 Key Laboratory of Agricultural Animal Genetics, Breeding and Reproduction of Ministry of Education, College of Animal Science and Technology, Huazhong Agricultural University, Wuhan 430070, China; 80206@hnuahe.edu.cn; 2 College of Animal Science and Technology, Henan University of Animal Husbandry and Economy, Zhengzhou 450046, China; 240320181102@stu.haust.edu.cn (L.G.); bl_lu232023@163.com (B.L.); liruiting210@163.com (R.L.); ny20071108@163.com (Y.N.); lqq17303946808@163.com (Q.L.); 17698238758@163.com (Y.Z.); wmr19263773243@163.com (M.W.); wb4117262007@163.com (B.W.); ypk323727@163.com (P.Y.); xinlei_king@163.com (X.W.); 3 College of Animal Science and Technology, Henan University of Science and Technology, Luoyang 471023, China

**Keywords:** European meat pigeon, Shiqi pigeon, duodenum, gut microbiota, growth traits

## Abstract

The duodenum is a key site of nutrient digestion and absorption in meat pigeons, and its microbial community may be associated with host body weight and breast muscle development. In this study, the duodenal microbiota of European meat pigeons and Shiqi pigeons at 14 and 28 days of age were analyzed using 16S rRNA gene sequencing. At 14 days of age, *Firmicutes* abundance was significantly higher in Shiqi pigeons than in European meat pigeons, whereas *Proteobacteria* showed the opposite pattern. *Lactobacillus* constituted the most abundant microbial group in both breeds. Spearman correlation analysis showed that *Firmicutes* and *Lactobacillus* were positively correlated with body weight and pectoral muscle percentage at 14 days of age, whereas *Proteobacteria* were negatively correlated with these traits. At 28 days of age, fewer between-breed differences in microbial community composition were detected; nevertheless, *Firmicutes* and *Lactobacillus* remained significantly positively correlated with body weight and pectoral muscle percentage in both breeds. These cross-sectional associations provide a preliminary reference for future studies of the duodenal microbiota and growth traits in meat pigeons.

## Simple Summary

Meat pigeons are economically important poultry in China. European meat pigeons and Shiqi pigeons differ markedly in growth rate and meat production performance. The duodenum is a key site for nutrient digestion and absorption, and its microbiota may be associated with host body weight and breast muscle development. This study compared the composition and PICRUSt2-predicted functional potential of the duodenal microbiota between the two breeds separately at 14 and 28 days of age and examined its associations with body weight and pectoral muscle percentage. Breed-related microbiota differences were more evident at 14 days of age, whereas fewer between-breed differences were detected at 28 days in this cross-sectional dataset. These findings provide a preliminary reference for further studies of duodenal microorganisms and microbiota-targeted intervention strategies in meat pigeons.

## 1. Introduction

China is the world’s largest producer and consumer of meat pigeons. Meat pigeon has become the fourth-largest poultry product after chicken, duck, and goose [1]. Among meat pigeon breeds, European meat pigeons and Shiqi pigeons differ significantly in growth performance. The Shiqi pigeon is a high-quality indigenous Chinese meat pigeon breed with strong adaptability and tolerance to coarse feed, and it weighs approximately 530 g at 28 days of age [2]. The European meat pigeon is a fast-growing, large-bodied breed developed by the French company Grimaud. It is characterized by rapid growth and high body weight, with squabs reaching a market weight of 600 g and a dressing percentage of approximately 87% [3]. The marked differences in growth rate and meat production between these breeds make them an ideal model for investigating the mechanisms that regulate growth and development in meat pigeons.

Gut microorganisms participate in nutrient metabolism and immune regulation, as reviewed in human studies [4]. In chickens, fecal microbiota transplantation has improved growth performance alongside changes in intestinal immune homeostasis [5]. Genetic, nutritional, and microbial factors have also been considered in reviews of skeletal muscle development in meat pigeons [6]. In other poultry, microbial composition has been associated with growth and carcass traits. In Yangzhou geese, 16S rRNA sequencing showed that the Firmicutes-to-Bacteroidetes ratio was approximately 20% higher in the high-body-weight group than in the low-body-weight group. At the genus level, the abundance of *Prevotellaceae_Ga6A1_group* was significantly positively correlated with leg and breast muscle weights [7]. Enterotype analysis in Muscovy ducks indicated that intestinal microbial community structure could serve as an indicator of growth performance and carcass traits [8]. Cecal microbial composition and functional potential also differ significantly between hybrid broilers and their parental lines, and non-additive microbial colonization patterns are associated with heterosis for breast muscle weight [9]. Other studies have shown that elevated lipopolysaccharide levels induced by dysbiosis disrupt muscle cell development by interfering with retinoic acid signaling [10], whereas *Megamonas* may affect lipid metabolism through propionate production [11]. Associations between intestinal microbiota and meat quality parameters, including muscle fiber characteristics and water-holding capacity, have also been reported in chickens [12]. In broilers, probiotics such as *Clostridium butyricum* promote the proliferation of beneficial bacteria and improve growth performance, immune responses, and intestinal barrier function [13]. In meat pigeons, gut microbial differences have been associated with fat deposition [14]. The proposed gut–muscle axis includes microbial metabolites that may influence muscle development and meat quality, although the evidence varies across experimental models [15]. Collectively, these findings indicate that gut microbial composition is associated with growth and carcass traits in poultry. However, most existing studies have focused on hindgut samples such as cecal contents and feces, whereas the foregut remains poorly characterized.

As the first segment of the small intestine, the duodenum is a major site of nutrient digestion and absorption and participates in pancreatic juice- and bile-mediated glucose, amino acid, and lipid metabolism. The duodenal microbial community can directly affect host nutrient utilization and thereby influence growth performance [16,17]. In poultry, duodenal microbial composition has been associated with variation in production traits, including egg production in Taihang chickens [16] and body weight in Xinghua chickens [18]. However, the relationships of the duodenal microbiota with breed-specific growth and breast muscle development in meat pigeons remain unclear. The age- and breed-associated variation in the duodenal microbiota and its associations with growth and development in different meat pigeon breeds have not been systematically investigated. Because the duodenum is directly involved in the initial absorption of amino acids required for skeletal muscle synthesis, differences in its microbial community structure may contribute to breed-specific differences in early growth and breast muscle development.

Most studies of the avian gut microbiota have focused on hindgut samples such as cecal contents and feces. Few have examined breed- and age-related differences in the duodenum, a key site of nutrient absorption. Comparative analyses of the duodenal microbiota of European meat pigeons and indigenous Shiqi pigeons and its associations with breast muscle development are also lacking. In this study, European meat pigeons and Shiqi pigeons of different ages were used as experimental animals. Live body weight and breast muscle weight were measured, and pectoral muscle percentage was calculated. High-throughput 16S rRNA gene sequencing was used to characterize duodenal microbial diversity and taxonomic composition. LEfSe (1.0) was used to identify differentially abundant microbial taxa between breeds, PICRUSt2 (v2.2.0-b) was used for COG and KEGG analyses, and Spearman correlation analysis was performed between microbial abundance and growth traits. We characterized the taxonomic composition and differentially abundant taxa in the duodenum of European meat pigeons and Shiqi pigeons at different developmental stages. We compared duodenal microbial community structure between breeds separately at each age, identified differential taxa, and predicted community functional profiles. This study provides preliminary insights into the structure and potential functions of the duodenal microbiota in different meat pigeon breeds. It also expands the basic knowledge of the intestinal microbiota of meat pigeons and provides a foundation for further research on their intestinal microecology.

## 2. Materials and Methods

### 2.1. Experimental Animals and Grouping

The European meat pigeons and Shiqi pigeons used in this experiment were provided by Henan Tianming Pigeon Industry Co., Ltd. (Zhengzhou, China). All squabs were raised under identical environmental conditions in the same facility, with controlled temperature (25 ± 2 °C), humidity (60 ± 5%), and a 14:10 light–dark cycle. Each breed was housed in separate but adjacent lofts with identical management practices, and all parent pigeons received the same commercial pigeon feed and had ad libitum access to water. A total of 15 breeding pairs were used for European meat pigeons and 15 breeding pairs for Shiqi pigeons. To avoid potential nest effects and pseudo-replication, squabs from different nests were randomly assigned to each sampling time point, with no more than one squab selected per nest for each age group, and no full siblings were included within the same age-breed combination. A total of 8 healthy male pigeons from each breed, hatched on the same day, were selected at 0, 14, and 28 days of age. These time points were selected because Day 14 may represent the transition from exclusive crop milk to mixed feeding, potentially serving as an important period for gut microbiota establishment. Meanwhile, Day 28 appears to correspond to near-market age, when growth rates tend to stabilize and the microbiota might approach a more mature state. Collectively, these two time points likely cover the period of maximum growth rate in meat pigeons. The squabs were naturally reared by their parents without artificial supplementary feeding, and farm management strictly followed standardized husbandry procedures throughout the experiment. On each sampling day, squabs of the corresponding age were transported to the laboratory together and allowed to acclimate before slaughter and sample collection.

### 2.2. Sample Collection and Processing

After transport, the squabs were allowed to acclimate for 30 min in a dark, quiet environment at a constant temperature to alleviate transportation stress. Each squab was then individually identified and weighed before being euthanized by jugular vein exsanguination. Slaughter performance was measured and calculated based on standard poultry production performance metrics (NY/T 823-2020 [19]) and measurement statistics. The bilateral breast muscles were removed and weighed. Pectoral muscle percentage was calculated as follows: pectoral muscle percentage (%) = (breast muscle weight/live body weight) × 100%. All weighing and anatomical dissections were performed by the same operator using a single-blind procedure. The duodenum, extending from the gastric pylorus to the duodenojejunal flexure, was isolated and excised. Each segment was opened longitudinally with a sterile scalpel, and the contents adhering to the luminal wall were gently scraped into sterile centrifuge tubes. Samples were immediately frozen in liquid nitrogen and then stored at −80 °C for subsequent 16S rRNA gene sequencing. Newly hatched 0-day-old squabs consumed only crop milk from their parents and had a highly immature intestine. Because the amount of contents in the duodenum was extremely limited, sufficient microbial samples could not be collected for sequencing. This study was approved by the Animal Experiment Ethics Committee of Henan University of Animal Husbandry and Economy (approval no. HNUAHESER2406k0301).

### 2.3. DNA Extraction and 16S rRNA Gene Sequencing

Because the amount of duodenal content from 0-day-old squabs was too small to yield sufficient high-quality DNA for library construction and sequencing, only samples collected at 14 and 28 days of age were included in subsequent analyses. A total of 6 biological replicates were randomly selected from the 8 individuals in each group for 16S rRNA gene sequencing. Total DNA was extracted using a commercial DNA extraction kit (TIANGEN Biotech, Beijing, China) according to the manufacturer’s instructions. DNA quality and integrity were assessed using a NanoDrop 2000 spectrophotometer (Thermo Fisher Scientific, Waltham, MA, USA) and 1% agarose gel electrophoresis; samples with A260/A280 ratios of 1.8–2.0 were considered acceptable. Qualified DNA was used as the template for PCR amplification of the V3-V4 region of the 16S rRNA gene with the universal primers 338F (5′-ACTCCTACGGGAGGCAGCAG-3′) and 806R (5′-GGACTACHVGGGTWTCTAAT-3′). Amplicons that passed quality assessment by 2% agarose gel electrophoresis were purified and used to construct sequencing libraries. The final sample set comprised 5 14-day-old Shiqi pigeons (1 sample was excluded because of inadequate DNA quality), 6 14-day-old European meat pigeons, 6 28-day-old Shiqi pigeons, and 6 28-day-old European meat pigeons. The libraries were subjected to paired-end sequencing on the DNBSEQ platform (MGI Tech, Shenzhen, China). Negative controls, including an extraction blank (sterile PBS processed without sample) and a PCR no-template control, were included in each sequencing batch. No microbial reads were detected above background threshold in the negative controls, confirming the absence of significant reagent or laboratory contamination.

### 2.4. Analysis of 16S rRNA Gene Sequencing Data

Raw data generated on the DNBSEQ platform were subjected to quality control to obtain clean data. A 25 bp sliding window was used, and trailing bases were removed when the mean quality score within the window was below 20. Reads that were too short, contained adapters or ambiguous bases, or had low complexity were discarded. Paired-end reads were merged into tags spanning the hypervariable region using FLASH (v1.2.11) with a minimum overlap of 15 bp and maximum mismatch rate of 0.1. The merged tags were then clustered into operational taxonomic units (OTUs) at 97% sequence similarity using USEARCH (v7.0.1090) via the UPARSE algorithm. Chimeric sequences were identified and removed using UCHIME (v4.2.40) by comparison against the gold database (v20110519). Representative sequences of OTUs were taxonomically classified against the SILVA database (Release 132) using the RDP classifier with a confidence threshold of 0.8. All quality-filtered tags were mapped back to OTU representative sequences using the usearch_global method to generate the OTU abundance table for each sample. Prior to diversity analysis, the OTU table was rarefied to 30,000 sequences per sample. OTU Rank Curves were generated in R (v3.4.1) to characterize taxon richness and evenness in each sample. Alpha diversity was assessed using the ACE and Chao1 indices for microbial richness and the Shannon and Simpson indices for microbial diversity. Specifically, Alpha diversity indices (Chao1, ACE, Shannon, and Simpson) were compared using Wilcoxon rank-sum tests with Benjamini–Hochberg FDR correction. Simpson index was calculated as D (where lower values indicate greater diversity). Beta diversity was analyzed using PERMANOVA on Bray–Curtis dissimilarity matrices with 999 permutations. Beta diversity was analyzed with ggplot in R (v3.4.1). Between-breed comparisons of relative abundance at the phylum and genus levels were performed using Wilcoxon rank-sum tests. The top 10 most abundant taxa were selected for analysis, and the mean relative abundance of each group is presented along with the significance of the differences, without FDR correction. Linear discriminant analysis effect size (LEfSe; LDA score > 2, *p* < 0.05) was used to identify potential biomarkers associated with age and breed, with the Wilcoxon rank-sum test applied for pairwise comparisons. Taxa were labeled with prefixes denoting taxonomic rank: p_, phylum; c_, class; o_, order; f_, family; and g_, genus. Microbial functions were predicted using PICRUSt2 in combination with R (v3.4.1), yielding predicted abundances and pathway annotations for Kyoto Encyclopedia of Genes and Genomes (KEGG) and Clusters of Orthologous Groups of proteins (COG) functions. Differences were evaluated based on both uncorrected *p*-values and FDR-adjusted *p*-values (q-values). Finally, Spearman correlation analysis was used to assess relationships between variables in separate subgroups, stratified by breed (European meat pigeons and Shiqi pigeons) and by age (14- and 28-day-old squabs) (*p* < 0.05). Multiple-testing correction was performed using the Benjamini–Hochberg false discovery rate (FDR) method, and correlations were visualized in Python 3.x (3.11) using scipy (1.13.1), pandas (2.2.2), and matplotlib (3.9.2).

### 2.5. Statistical Analysis

Growth performance was analyzed using a two-way analysis of variance (ANOVA) framework with Age, Breed, and their interaction as fixed effects. Body weight and pectoral muscle percentage were measured at three developmental stages: day 0, 14, 28. Data were processed by trimming extreme values from each breed × age group, resulting in *n* = 6 per group. Pairwise comparisons between European and Shiqi breeds at each time point were conducted using independent samples *t*-tests, with effect sizes reported as Hedges’ g (small-sample corrected standardized mean difference) accompanied by 95% confidence intervals. All analyses were performed using Python 3.x with appropriate statistical packages. Data were presented as the mean ± standard error of the mean (mean ± SEM). * indicated *p* < 0.05, ** indicated *p* < 0.01, *** indicated *p* < 0.001, and ns indicated no significant difference between groups (*p* > 0.05).

## 3. Results

### 3.1. Comparison of Body Weight and Breast Muscle Development Between European Meat Pigeons and Shiqi Pigeons at Different Ages

To characterize differences in growth performance between European meat pigeons and Shiqi pigeons, body weight and pectoral muscle percentage were measured at 0, 14, and 28 days of age. A significant Age × Breed interaction was observed for both body weight (F(2,30) = 8.47, *p* < 0.001) and breast muscle percentage (F(2,30) = 6.23, *p* = 0.005), indicating that the two breeds exhibit fundamentally distinct growth patterns across measured time points rather than a simple constant offset (Figure 1).

At 0 days of age, body weight was similar between European meat pigeons (E) and Shiqi pigeons (S), with no significant difference (Δ = +0.5 g, 95% CI [−0.3, +1.3], g = +0.75; *p* > 0.05; Figure 2A). Thus, the two breeds had comparable hatch weights. At 14 days of age, European meat pigeons had a significantly higher body weight than Shiqi pigeons (Δ = +48.6 g, 95% CI [+12.2, +85.0], g = +1.59; *p* < 0.05; Figure 2B). At 28 days of age, their body weight remained significantly higher (Δ = +53.7 g, 95% CI [+9.7, +97.6], g = +1.45; *p* < 0.05; Figure 2C). Body weight increased continuously with age in both breeds. Pectoral muscle percentage also differed between the breeds across ages. At 0 days of age, Shiqi pigeons had a significantly higher pectoral muscle percentage than European meat pigeons (Δ = −0.71 pp, 95% CI [−0.99, −0.43], g = −3; *p* < 0.001; Figure 2D), indicating a greater proportional development of breast muscle immediately after hatching. At 14 days of age, pectoral muscle percentage was significantly higher in European meat pigeons than in Shiqi pigeons (Δ = +1.49 pp, 95% CI [+0.71, +2.27], g = +2.28; *p* < 0.01; Figure 2E). By 28 days of age, pectoral muscle percentages had become similar and no longer differed significantly between the breeds (Δ = +1.01 pp, 95% CI [−0.52, +2.54], g = +0.79; *p* > 0.05; Figure 2F). Pectoral muscle percentage increased with age in both breeds. Thus, European meat pigeons had a lower pectoral muscle percentage than Shiqi pigeons at 0 days of age, surpassed them by 14 days of age, and showed no between-breed difference at 28 days of age. Effect sizes and 95% confidence intervals are provided in the Supplementary Materials (Table S1).

### 3.2. Sequencing Data Quality Analysis

After quality control filtering, the duodenal microbial samples yielded 1,614,767 valid reads, with an average of 70,207 reads per sample. The total amount of clean data was 968.86 Mb, averaging 42.12 Mb per sample, and the data utilization rate was approximately 99.3% (Table S2). USEARCH (v7.0.1090) was used to cluster the merged tags into OTUs. Sequences with greater than 97% similarity were clustered into OTUs. All tags were mapped back to representative OTU sequences using usearch-global to obtain an OTU abundance table for each sample (Table S3). The OTU Rank Curves (Figure 3) showed that species richness (horizontal span) and evenness (curve smoothness) were comparable across samples.

### 3.3. Diversity Analysis of the Duodenal Microbiota in European Meat Pigeons and Shiqi Pigeons

To characterize microbial differences between the two breeds during the brooding period, alpha diversity of the duodenal microbiota was analyzed at 14 and 28 days of age. At 14 days of age, the Chao1, ACE, Shannon, and Simpson indices did not differ significantly between European meat pigeons and Shiqi pigeons (*p* > 0.05; Figure 4A). By 28 days of age, alpha diversity differed significantly between the breeds. The Chao1 and ACE indices were significantly higher in European meat pigeons than in Shiqi pigeons (*p* < 0.05), the Shannon index was also significantly higher (*p* < 0.01), and the Simpson index (D) was significantly lower in European meat pigeons (*p* < 0.05; Figure 4B). These results indicated that duodenal microbial richness and diversity were similar between the breeds at 14 days of age. At 28 days of age, the indices indicated greater taxon richness and diversity in European meat pigeons than in Shiqi pigeons.

Beta diversity analysis showed a highly significant difference in the duodenal microbiota between European meat pigeons and Shiqi pigeons at 14 days of age (*p* < 0.001; Figure 5A). At 28 days of age, no significant between-breed difference in community structure was detected (*p* > 0.05; Figure 5B).

### 3.4. Relative Abundance of the Duodenal Microbiota at the Phylum and Genus Levels in European Meat Pigeons and Shiqi Pigeons

The relative abundance of duodenal microbial taxa was analyzed at the phylum and genus levels in European meat pigeons and Shiqi pigeons at 14 and 28 days of age (Figure 6). At the phylum level, *Firmicutes* was the most abundant phylum in both breeds at 14 days of age, followed by *Bacteroidota*, *Proteobacteria*, and other phyla (Figure 6A). At 28 days of age, *Firmicutes* remained predominant, whereas the abundances of the other phyla were extremely low (Figure 6B). At the genus level, *Lactobacillus* was the most abundant genus in both breeds at 14 days of age, followed by *Pseudomonas*, *Burkholderia*, and a few other genera (Figure 6C). *Lactobacillus* remained predominant in both breeds at 28 days of age (Figure 6D). The phylum- and genus-level results consistently showed differences in duodenal microbial composition between the breeds at 14 days of age. At 28 days of age, *Firmicutes* and *Lactobacillus* were the dominant taxa in both breeds, and fewer between-breed differences in relative abundance were detected.

At 14 days of age, the relative abundance of *Firmicutes* was significantly higher in Shiqi pigeons than in European meat pigeons (S, 95.42%; E, 76.98%; *p* < 0.05). In contrast, the relative abundance of *Proteobacteria* was significantly higher in European meat pigeons than in Shiqi pigeons (E, 14.94%; S, 0.5%; *p* < 0.01). The relative abundance of *Lactobacillus* did not differ significantly between the breeds at either 14 or 28 days of age (14 days: S, 94.70% and E, 76.40%; 28 days: S, approximately 95% and E, 97%; *p* > 0.05). Other genera differed between the breeds. At 14 days of age, the relative abundances of *Burkholderia-Caballeronia-Paraburkholderia*, *Pseudomonas*, *Ralstonia*, and *Sphingomonas* were significantly higher in European meat pigeons than in Shiqi pigeons (*p* < 0.05). Most of these genera belong to *Proteobacteria*, consistent with the higher phylum-level abundance of *Proteobacteria* in European meat pigeons. *Aeriscardovia* was significantly more abundant in Shiqi pigeons (*p* < 0.05). By 28 days of age, the number of differentially abundant genera had decreased markedly. Only *Aeriscardovia* was significantly more abundant in European meat pigeons (*p* < 0.05), although its relative abundance was extremely low (approximately 1%) (Supplementary Figure S1).

### 3.5. LEfSe Analysis of the Duodenal Microbiota in European Meat Pigeons and Shiqi Pigeons

LEfSe analysis (LDA threshold > 2) was used to identify microbial taxa at different taxonomic levels that differed significantly in abundance between the breeds at each age. At 14 days of age, several differentially abundant microbial taxa were identified. The characteristic taxa in the duodenum of European meat pigeons were mainly *Proteobacteria* and members of the families *Burkholderiaceae* and *Pseudomonadaceae*. In Shiqi pigeons, the principal characteristic taxa were *Firmicutes*, *Lactobacillus*, *Lactobacillaceae*, and the genus *Aeriscardovia* within *Bifidobacteriaceae* of *Actinobacteriota* (Figure 7A). At 28 days of age, the number of differential microbial taxa between the breeds decreased markedly, and community differentiation was greatly attenuated. The characteristic taxa in the duodenum of European meat pigeons were *Actinobacteriota* and taxa related to *Bifidobacteriaceae*, whereas *Staphylococcus* was enriched in Shiqi pigeons (Figure 7B).

Although the overall relative abundance of Lactobacillus did not differ significantly between breeds (Wilcoxon rank-sum test, *p* > 0.05), LEfSe identified it as a discriminatory biomarker (LDA > 2) in Shiqi pigeons at 14 days. This apparent discrepancy arises because LEfSe captures subtle but consistent abundance patterns across the full taxonomic hierarchy, whereas a pairwise non-parametric test on a single highly dominant genus may lack power to detect small proportional differences. Given the compositional nature of 16S data and the small sample size, LEfSe results should be regarded as exploratory and hypothesis-generating.

### 3.6. COG and KEGG Analyses of the Duodenal Microbiota in European Meat Pigeons and Shiqi Pigeons

It should be noted that the COG and KEGG functional profiles presented below were inferred from 16S rRNA gene sequences using PICRUSt2 and represent predicted metabolic potential rather than directly measured gene expression or metabolic activity. These predictions require validation through shotgun metagenomics or metabolomics.

PICRUSt2-based COG functional prediction showed that the microbiota of 14-day-old European meat pigeons was significantly enriched in intracellular trafficking and secretion, defense mechanisms, mobilome functions, and extracellular structures (*p* < 0.01; Figure 8A). In contrast, the microbiota of Shiqi pigeons was significantly enriched in basic information-processing functions, including translation, ribosomal structure, and transcription (*p* < 0.05; Figure 8A). At 28 days of age, differences in the relative abundance of COG functions between the breeds had decreased. The absolute log2 fold changes between groups were markedly lower, only a few pathways showed weak differences in enrichment, and adjusted *p*-values for most functions approached 1. Thus, the differentiation of microbial metabolic functions between the breeds had largely disappeared. The microbiota of Shiqi pigeons was significantly enriched in posttranslational modification and chaperones and in lipid transport and metabolism (*p* < 0.05; Figure 8B), and it was highly enriched in mobilome functions (*p* < 0.01; Figure 8B). Nucleotide transport and metabolism were more enriched in European meat pigeons (*p* < 0.05; Figure 8B). These findings indicated that 14 days of age was a key stage for functional differentiation of the duodenal microbial community in meat pigeons. As the pigeons aged to 28 days, statistical power to detect breed-specific functional differences diminished substantially at 28 days.

PICRUSt2-based KEGG pathway enrichment analysis showed significant differentiation of duodenal microbial metabolic pathways between the breeds at 14 days of age. Cell motility and signal transduction were highly enriched in European meat pigeons (*p* < 0.01; Figure 9A), and membrane transport and environmental adaptation were also significantly enriched (*p* < 0.05; Figure 9A). In contrast, only the translation pathway was significantly enriched in the microbiota of Shiqi pigeons (*p* < 0.05; Figure 9A). At 28 days of age, differences in the relative abundance of KEGG pathways between the breeds had decreased markedly. Only the metabolism of cofactors and vitamins was significantly enriched in the microbiota of European meat pigeons, whereas signal transduction was significantly enriched in that of Shiqi pigeons (*p* < 0.05; Figure 9B). KEGG pathway analysis showed that breed-related differences in the metabolic functions of the duodenal microbiota decreased substantially with age. This pattern was consistent with the COG predictions and the reduced between-breed differences in microbial taxonomic composition.

### 3.7. Associations of the Duodenal Microbiota with Body Weight and Pectoral Muscle Percentage

Spearman correlation analysis was used to assess associations between key differential taxa and live body weight (BW) and pectoral muscle percentage (PMP) in squabs, pooling both breeds within each age (14 days, *n* = 11; 28 days, *n* = 12). At 14 days of age, *p_Firmicutes* and *g_Lactobacillus* were strongly and significantly positively correlated with both BW and PMP. *p_Proteobacteria* was significantly negatively correlated with BW, *g_Burkholderia-Caballeronia-Paraburkholderia* was strongly and significantly negatively correlated with BW, and *g_Ralstonia* was significantly negatively correlated with PMP. At 28 days of age, *p_Actinobacteriota* and *g_Aeriscardovia* were significantly negatively correlated with both BW and PMP (Figure 10).

Spearman correlation analysis was performed separately for each breed, pooling both age groups (14 and 28 days) within breed, to assess associations between key differential taxa and growth traits. For European meat pigeons (*n* = 12), seven taxa showed significant correlations: *g_Lactobacillus* and *p_Firmicutes* were positively correlated with both BW and PMP, while *g_Sphingomonas*, *g_Pseudomonas*, *g_Burkholderia-Caballeronia-Paraburkholderia*, *g_Ralstonia*, and *p_Proteobacteria* were negatively correlated with growth traits. For Shiqi pigeons (n = 11), five taxa exhibited significant associations: *g_Lactobacillus* and *p_Firmicutes* were positively correlated with both BW and PMP, while *g_Pseudomonas*, *g_Burkholderia-Caballeronia-Paraburkholderia*, and *g_Veillonella* were negatively correlated with growth performance. Both breeds shared common associations with *g_Lactobacillus* and *p_Firmicutes* (positive), and *g_Pseudomonas* and *g_Burkholderia-Caballeronia-Paraburkholderia* (negative), suggesting conserved microbiota–growth relationships across breeds (Figure 11).

## 4. Discussion

This study compared the duodenal microbiota of European meat pigeons and Shiqi pigeons at 14 and 28 days of age. Breed-related differences in taxonomic composition and PICRUSt2-predicted functional profiles were more apparent at 14 days of age, whereas fewer between-breed differences were detected at 28 days in these age-stratified cross-sectional comparisons. These observations provide a preliminary basis for further investigation of relationships between the duodenal microbiota and growth traits.

In both poultry and mammals, early postnatal life is a window of rapid microbial colonization and fluctuation in taxonomic composition. The microbiota subsequently develops into a relatively stable, mature community. In laying hens, the fecal microbiota undergoes stage-specific restructuring and gradually stabilizes with age [20]. In pigeons, the intestinal microbial community of White King squabs changes significantly across ages, and the first 48 h after hatching are suggested to be a critical period for microbial maturation [21]. In the present study, OTU Rank Curves indicated notable differences in duodenal microbial richness and evenness between age points; however, given our cross-sectional design, attribution of these patterns to specific factors requires caution. Breed effects on community structure were more pronounced at 14 days compared to 28 days. In the present study, alpha diversity indices did not differ significantly between breeds at 14 days. At 28 days, European meat pigeons had higher Chao1, ACE, and Shannon indices and a lower Simpson index (D), consistent with greater richness and diversity. No significant between-breed difference was detected in the reported beta diversity analysis at 28 days. These findings are not necessarily inconsistent: alpha diversity describes richness and the distribution of abundance within a sample, whereas beta diversity describes differences in composition between samples.

At the phylum level, *Firmicutes* was overwhelmingly dominant in the duodenum of both breeds. Its abundance increased with age, whereas *Proteobacteria* decreased concurrently. This pattern is consistent with the progressive establishment of an anaerobic intestinal environment in poultry and the developmental succession from facultative to obligate anaerobes [22]. *Firmicutes* can degrade starch to produce short-chain fatty acids, which supply energy to the intestinal epithelium and serve as substrates for de novo lipogenesis, thereby promoting energy deposition and the growth of muscle and adipose tissues [23]. An increase in *Proteobacteria* is generally regarded as a marker of dysbiosis. As facultative anaerobes, these bacteria can proliferate rapidly when the anaerobic environment is disrupted, thereby damaging the intestinal barrier and increasing energy expenditure [24,25]. In the present study, the relative abundance of *Proteobacteria* was significantly higher in European meat pigeons than in Shiqi pigeons at 14 days of age, although European meat pigeons also had a significantly higher body weight. The taxa enriched in European meat pigeons were common environmental groups, including *Burkholderiaceae* and *Pseudomonadaceae*, whereas pathogens such as *Escherichia coli* and *Salmonella* were not detected. Their enrichment represented temporary dominance by facultative anaerobes before the anaerobic environment was fully established during early brooding. As anaerobic conditions developed, *Proteobacteria* declined by 28 days of age. The higher tolerance to *Proteobacteria* in fast-growing European meat pigeons at 14 days may reflect breed-specific physiological adaptations. Fast-growing breeds may have enhanced intestinal barrier function with tighter junctions and mucus integrity [26], more active innate immunity with antimicrobial peptides [27], and metabolic demands favoring facultative anaerobes that efficiently process nutrient-rich crop milk. However, the mechanisms require further investigation through comparative studies of barrier function, immune gene expression, and mucus properties. The body-weight advantage of European meat pigeons, a fast-growing breed selected for large body size, was determined mainly by genetic background [28]. Its coexistence with transient early enrichment of *Proteobacteria* indicates an association rather than a causal relationship. Nevertheless, validating these observations still necessitates the measurement of intestinal barrier function, inflammation, and absolute microbial load. At the genus level, *Lactobacillus* was overwhelmingly dominant in both breeds. It can promote lipid metabolism by upregulating genes involved in beta-oxidation, thermogenesis, and lipolysis and is a core beneficial taxon in the poultry intestine [29]. LEfSe showed clear differentiation in characteristic taxa between the breeds at 14 days of age. European meat pigeons were enriched in *Proteobacteria* members such as *Burkholderiaceae*, whereas Shiqi pigeons were enriched in *Lactobacillus* and *Aeriscardovia*. The marked reduction in differentially abundant genera between breeds at 28 days of age further confirms that age is a key driver of microbial community structure. These interpretations should be considered as hypotheses requiring functional validation through controlled intervention studies. Given compositional data and modest sample size, LEfSe results should be interpreted cautiously.

Differences in microbial taxonomic composition ultimately affect host nutrient metabolism and growth through microbial metabolic functions. COG and KEGG predictions showed that the microbiota of Shiqi pigeons was enriched in translation, ribosomal structure, and biosynthetic pathways at 14 days of age. Pathways such as translation reflect the growth rate and metabolic activity of the bacteria themselves [30]. Although translation pathways were enriched in Shiqi pigeons during the early stage, their body weight at 14 days of age was significantly lower than that of European meat pigeons. Zhang et al. [31] similarly found relative enrichment of ribosome- and translation-related genes in the jejunal microbiota of low-body-weight chickens. Thus, enrichment of these functions may reflect the proliferative requirements of the microbiota rather than a direct effect on host growth. In contrast, the microbiota of European meat pigeons was enriched in extracellular structures and cell motility during the early stage. These functions are largely related to bacterial appendages such as flagella and pili and to chemotactic colonization. They are essential for intestinal bacteria to colonize the host mucosa and participate in early community assembly [32,33], thereby facilitating rapid microbiota establishment and supporting the persistent early body-weight advantage of European meat pigeons. At 28 days of age, the microbiota of Shiqi pigeons displayed a trend towards enrichment in lipid transport and metabolism. Previous studies have shown that the microbiota of high-body-weight chickens contains more lipid metabolism genes and that fecal microbiota transplantation can alter abdominal fat deposition in recipient chickens [34]. The gut microbiota can also regulate intramuscular fat deposition through the bile acid–farnesoid X receptor axis [35]. This functional shift indicates a transition from microbial self-synthesis toward energy provision for the host. These divergent metabolic profiles likely reflect breed-specific growth strategies shaped by selective breeding. Shiqi pigeons at 28 days of age, may prioritize lipid storage for energy reserves, while European meat pigeons, bred for rapid muscle development, maintain amino acid metabolism to support continued protein synthesis. This metabolic divergence aligns with production traits: higher breast muscle yield in European meat pigeons versus greater fat deposition in Shiqi pigeons, reflecting breed-specific phenotypic outcomes. At 28 days of age, the microbiota of European meat pigeons displayed a trend towards enrichment in amino acid metabolism and the metabolism of cofactors and vitamins. These functions can provide substrates and nitrogenous metabolites for muscle protein deposition [36], consistent with their body-weight advantage at the later stage. COG and KEGG predictions provide hypotheses regarding microbial functional potential, but they do not measure microbial gene expression, metabolite production, or nutrient supply to the host. They merely serve as a rationale for measuring lipid handling and protein metabolism.

To further explore the potential associations between the duodenal microbiota and growth traits, Spearman correlation analyses were performed between differential taxa at the phylum and genus levels and both body weight and pectoral muscle percentage in 14- and 28-day-old pigeons. these associations differed notably between the two age groups. At 14 days of age, both *Firmicutes* and *Lactobacillus* showed significant and strong positive correlations with body weight and pectoral muscle percentage, and their functional roles have been elucidated previously [23,27]. These findings suggest that during the early brooding stage, acid-producing and fermentative microbial communities, represented by *Firmicutes* and *Lactobacillus*, are more fully established and are more significantly associated with muscle growth in squabs. Conversely, *Proteobacteria* and some of its members, such as *Burkholderia* and *Ralstonia*, exhibited significant negative correlations with all growth traits. By 28 days of age, the strong correlations involving *Firmicutes* and *Lactobacillus* were no longer significant; instead, *Actinobacteriota* and *Aeriscardovia* showed significant negative correlations with body weight and pectoral muscle percentage. These observations indicate that the core microbial markers associated with growth undergo dynamic shifts during the brooding period, suggesting that the contribution of the microbiota to host growth occurs vary across age groups. This study employed a breed-stratified network analysis approach by pooling age groups to systematically reveal the associations between duodenal microbiota and growth traits in meat pigeons. Three key findings emerged: First, *g_Lactobacillus* and *p_Firmicutes* were identified as core beneficial taxa shared by both breeds, exhibiting strong positive correlations with growth performance. Second, *p_Proteobacteria* and *g_Burkholderia-Caballeronia-Paraburkholderia* negatively impacted growth traits, highlighting the Firmicutes-to-Proteobacteria ratio as an important indicator for growth assessment. Third, breed-specific taxa were identified, including *g_Ralstonia* in European meat pigeons and *g_Veillonella* in Shiqi pigeons, suggesting that differentiated microbiota modulation strategies may be required for each breed.

In summary, these findings indicate distinct patterns of microbial colonization between the two breeds during early brooding, corresponding to their divergent growth performance. Shiqi pigeons tended to establish a robust, acid-producing fermentative community rapidly, whereas European meat pigeons tended to support their superior phenotype through enhanced colonization and environmental adaptation. Unlike previous studies of the cecum and feces, the present study focused on the duodenum, the initial site of nutrient absorption. Breed-related differences in the microbiota of this region were likewise concentrated in the early brooding stage and were associated with body weight and pectoral muscle percentage. Therefore, the role of the foregut microbiota in early growth should not be overlooked. Sampling duodenal and cecal contents from the same individuals in a future study would allow a paired comparison while accounting for breed, age, and nest. Combining this design with metabolite measurements would help determine whether associations with growth are shared across intestinal regions or specific to the duodenum.

## 5. Conclusions

This study employed 16S rRNA gene sequencing to conduct a preliminary, age-stratified cross-sectional comparison of the duodenal microbiota in European meat pigeons and Shiqi pigeons at 14 and 28 days of age. Greater between-breed taxonomic and predicted functional differences were detected at 14 days compared to 28 days, with effect sizes being larger at the earlier time point. At 14 days, *Firmicutes* and *Lactobacillus* abundance showed positive correlations with body weight and pectoral muscle percentage, while distinct (predominantly negative) correlations involving *Actinobacteriota* and *Aeriscardovia* were observed at 28 days. These observations should be interpreted as exploratory findings, given our cross-sectional design and modest sample size. They provide a foundational reference for future longitudinal studies with repeated individual sampling and microbiota-targeted intervention trials.

## Figures and Tables

**Figure 1:**
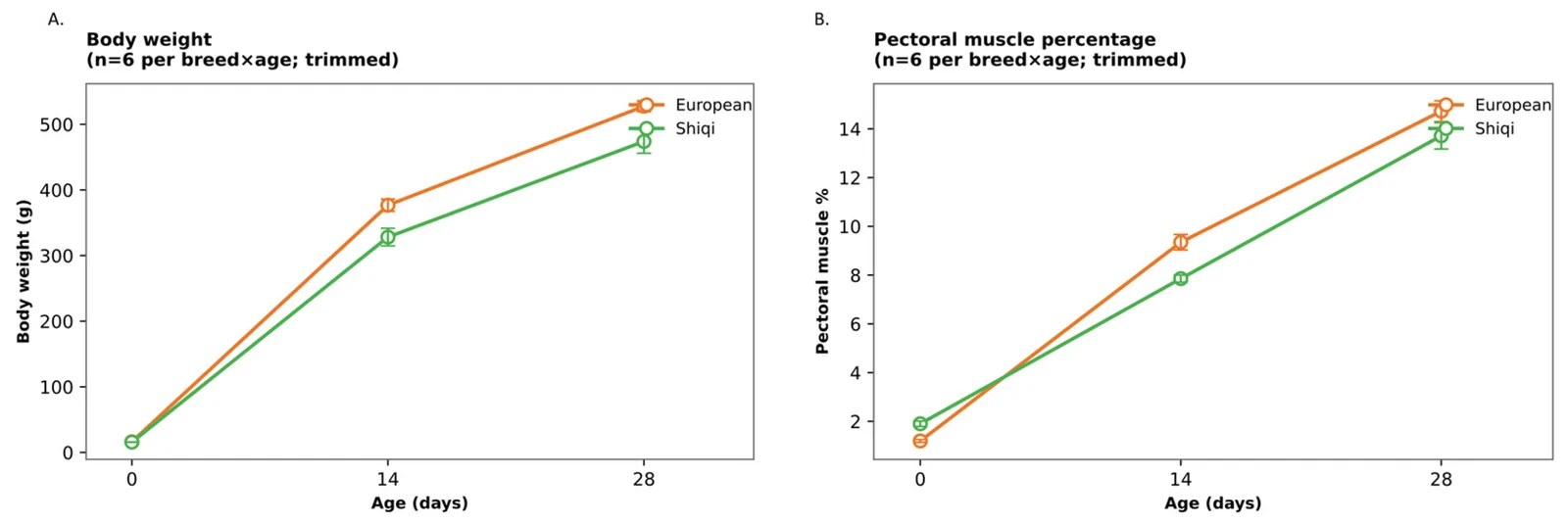
Growth trajectories of European and Shiqi squabs at different ages. (**A**) Body weight; (**B**) Pectoral muscle percentage.

**Figure 2:**
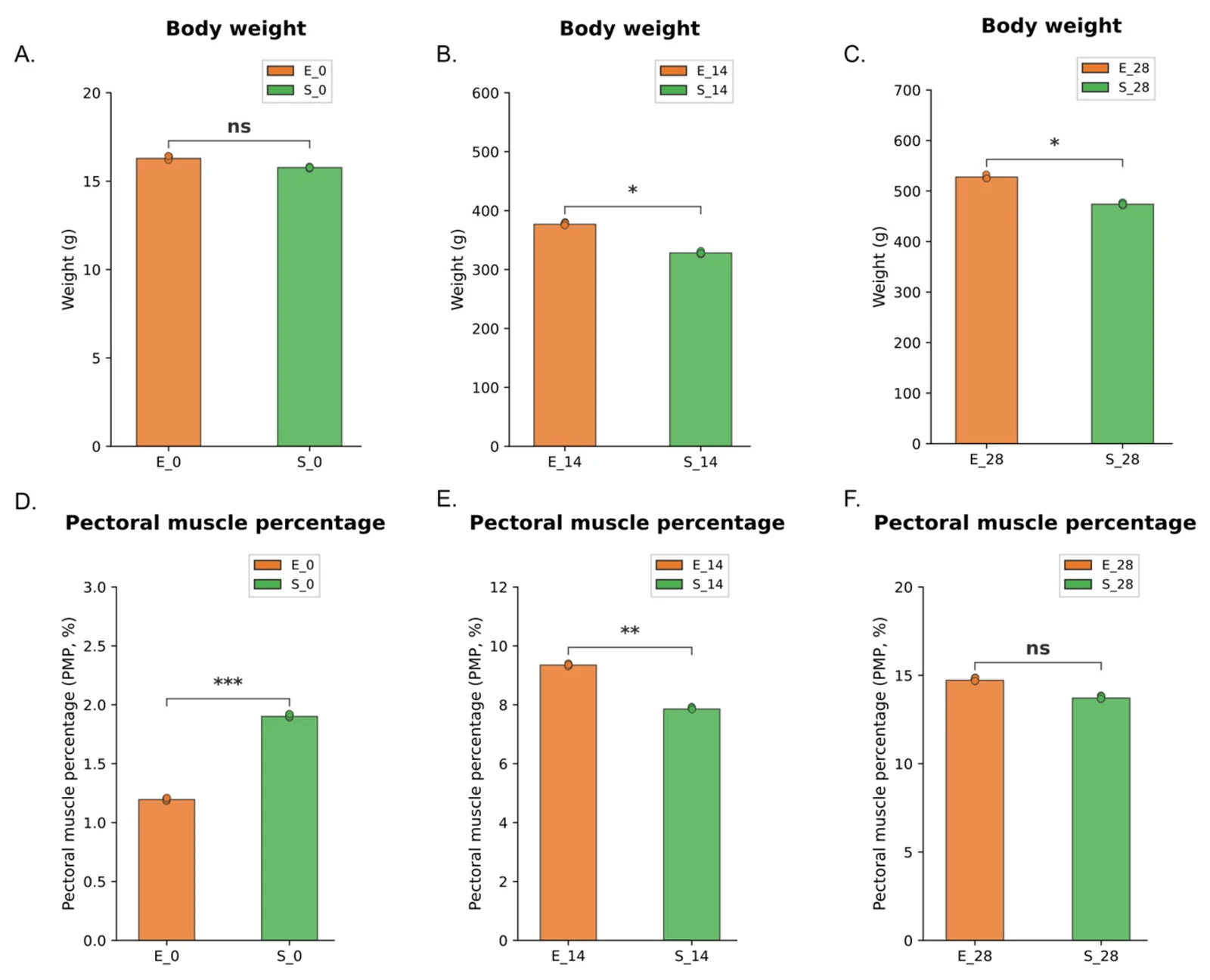
Comparison of body weight and breast muscle development between European meat pigeons and Shiqi pigeons at different ages. (**A**–**C**) Body weight of the two breeds at 0, 14, and 28 days of age, respectively. (**D**–**F**) Pectoral muscle percentage of the two breeds at 0, 14, and 28 days of age, respectively. E, European meat pigeon; S, Shiqi pigeon; Weight, body weight; PMP, pectoral muscle percentage. * *p* < 0.05, ** *p* < 0.01, *** *p* < 0.001; ns, not significant (*p* > 0.05).

**Figure 3:**
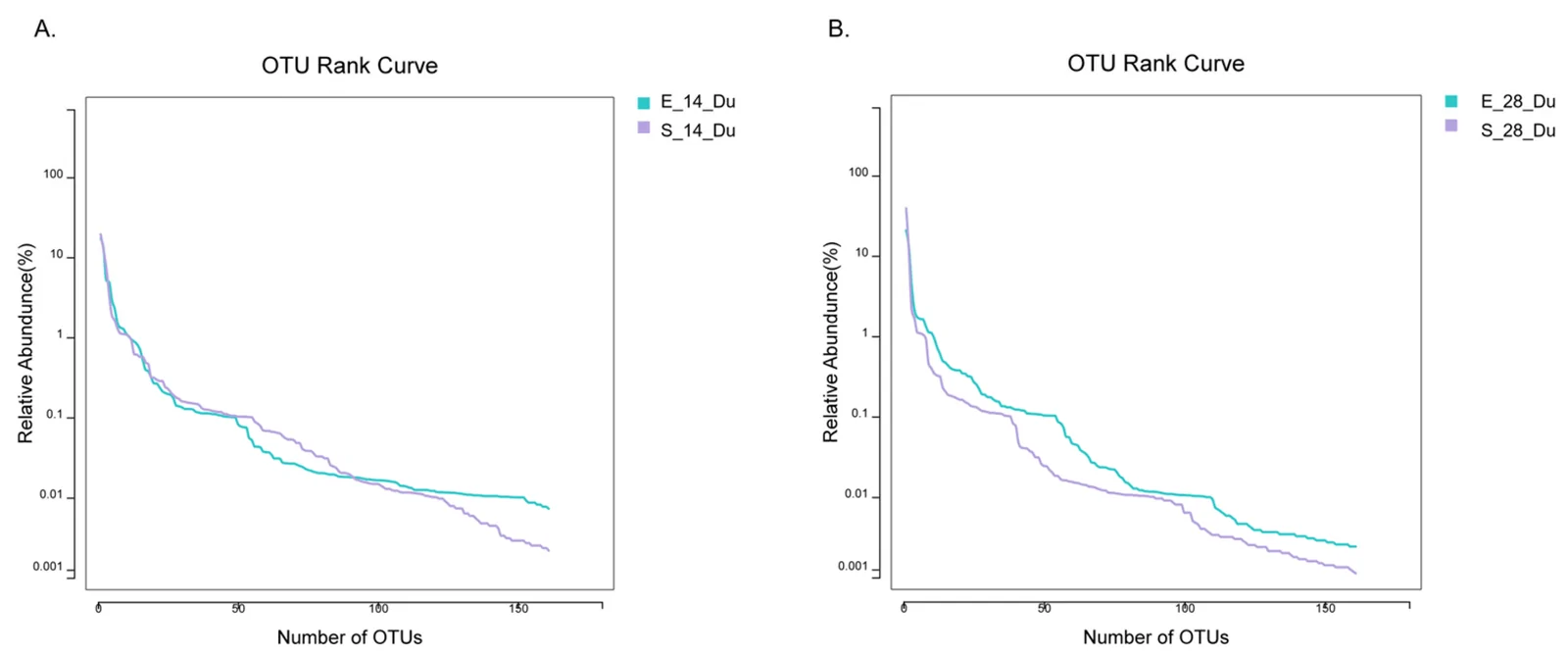
OTU Rank Curves of the duodenal microbiota of European meat pigeons and Shiqi pigeons. (**A**) OTU rank-abundance curves for the two breeds at 14 days of age. (**B**) OTU rank-abundance curves for the two breeds at 28 days of age. Blue E_14 denoted European meat pigeons, and purple S_14 denoted Shiqi pigeons. The span of a curve along the horizontal axis reflected taxon richness, whereas curve smoothness reflected the evenness of taxon abundance.

**Figure 4:**
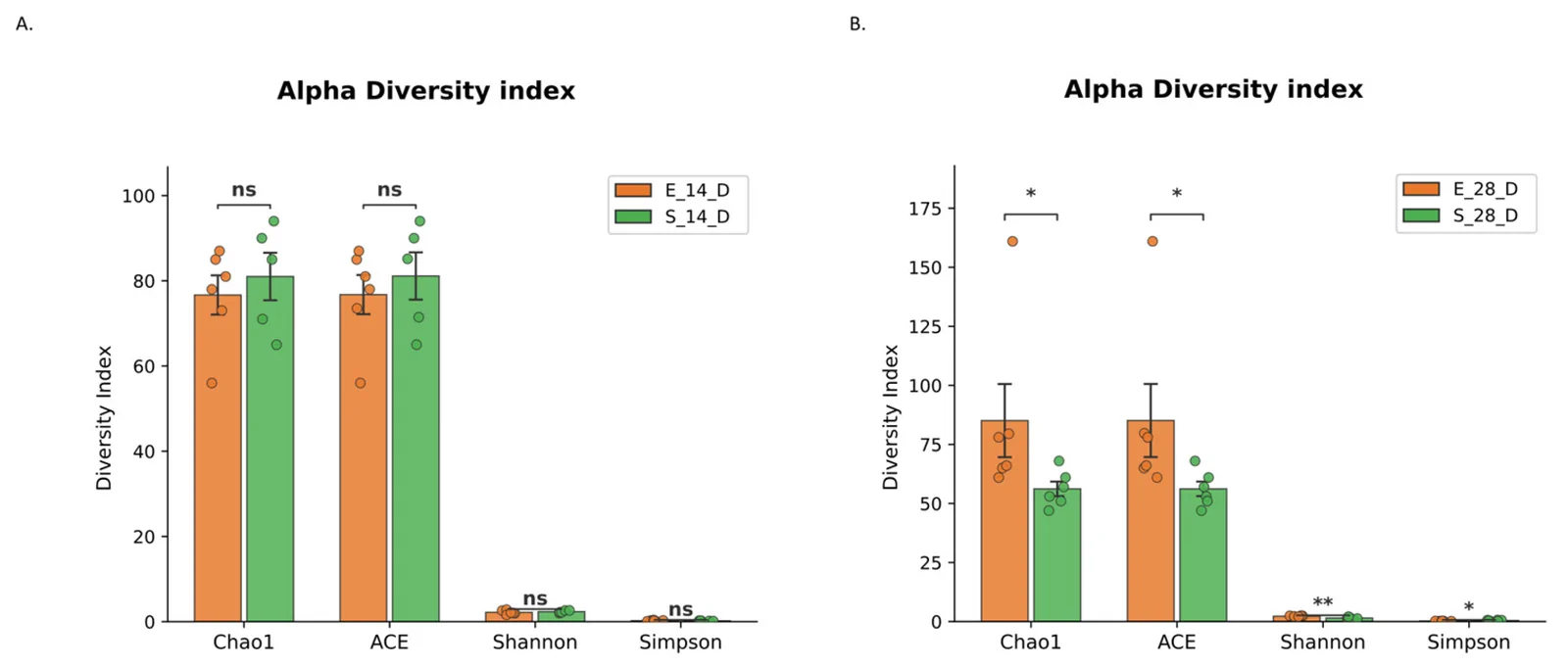
Alpha diversity analysis of the duodenal microbiota in European meat pigeons and Shiqi pigeons at different ages. (**A**) 14 days of age. (**B**) 28 days of age. Green bars (S_14/28_D) represent duodenal samples from 14- and 28-day-old Shiqi pigeons, and orange bars (E_14/28_D) represent duodenal samples from 14- and 28-day-old European meat pigeons. * *p* < 0.05, ** *p* < 0.01; ns, not significant (*p* > 0.05).

**Figure 5:**
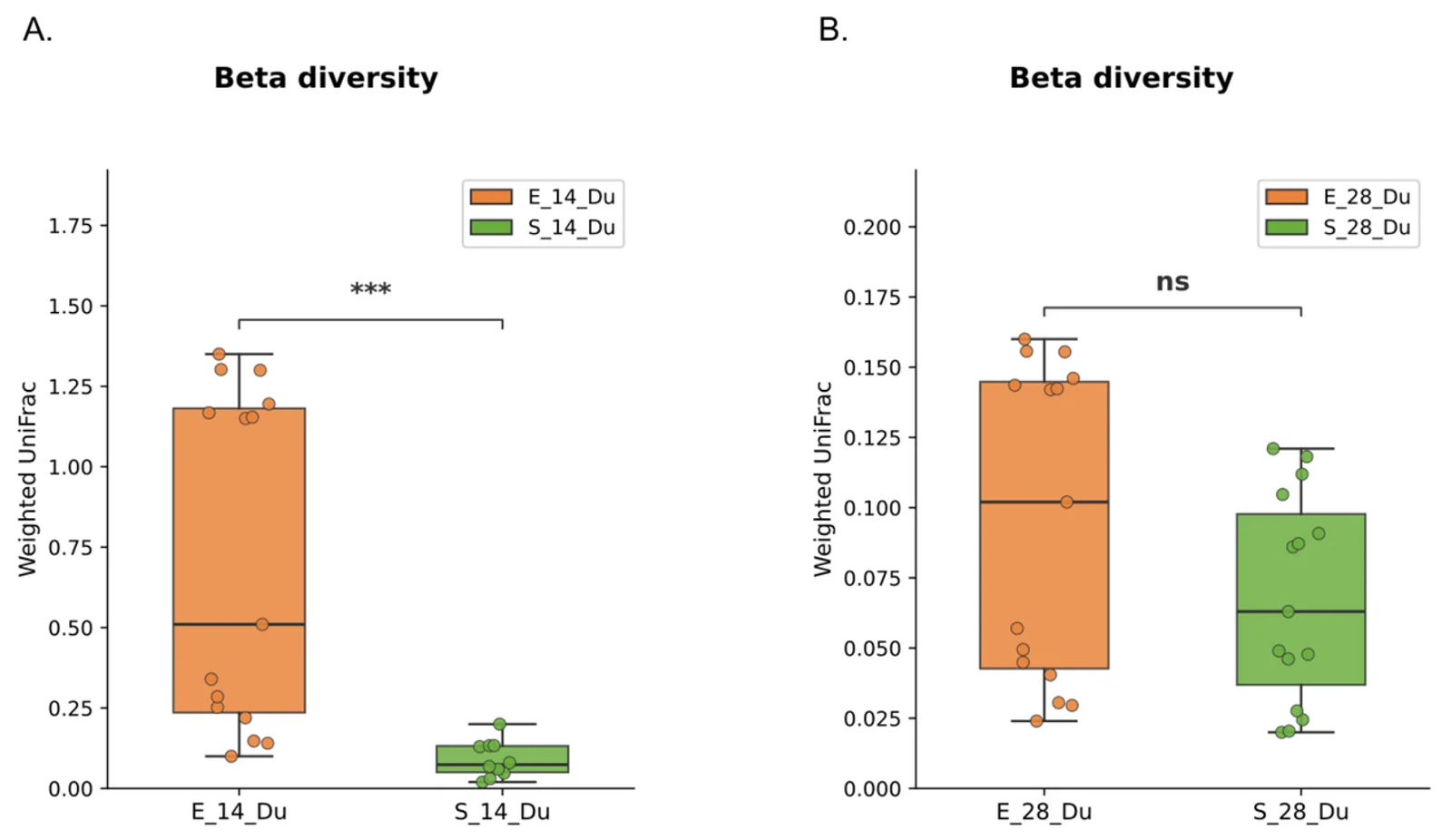
Beta diversity analysis of the duodenal microbiota in European meat pigeons and Shiqi pigeons at different ages. (**A**) 14 days of age. (**B**) 28 days of age. Green bars (S_14/28_D) represent duodenal samples from 14- and 28-day-old Shiqi pigeons, and orange bars (E_14/28_D) represent duodenal samples from 14- and 28-day-old European meat pigeons. From bottom to top, the 5 lines represented the minimum, first quartile, median, third quartile, and maximum, respectively. *** *p* < 0.001; ns, not significant (*p* > 0.05).

**Figure 6:**
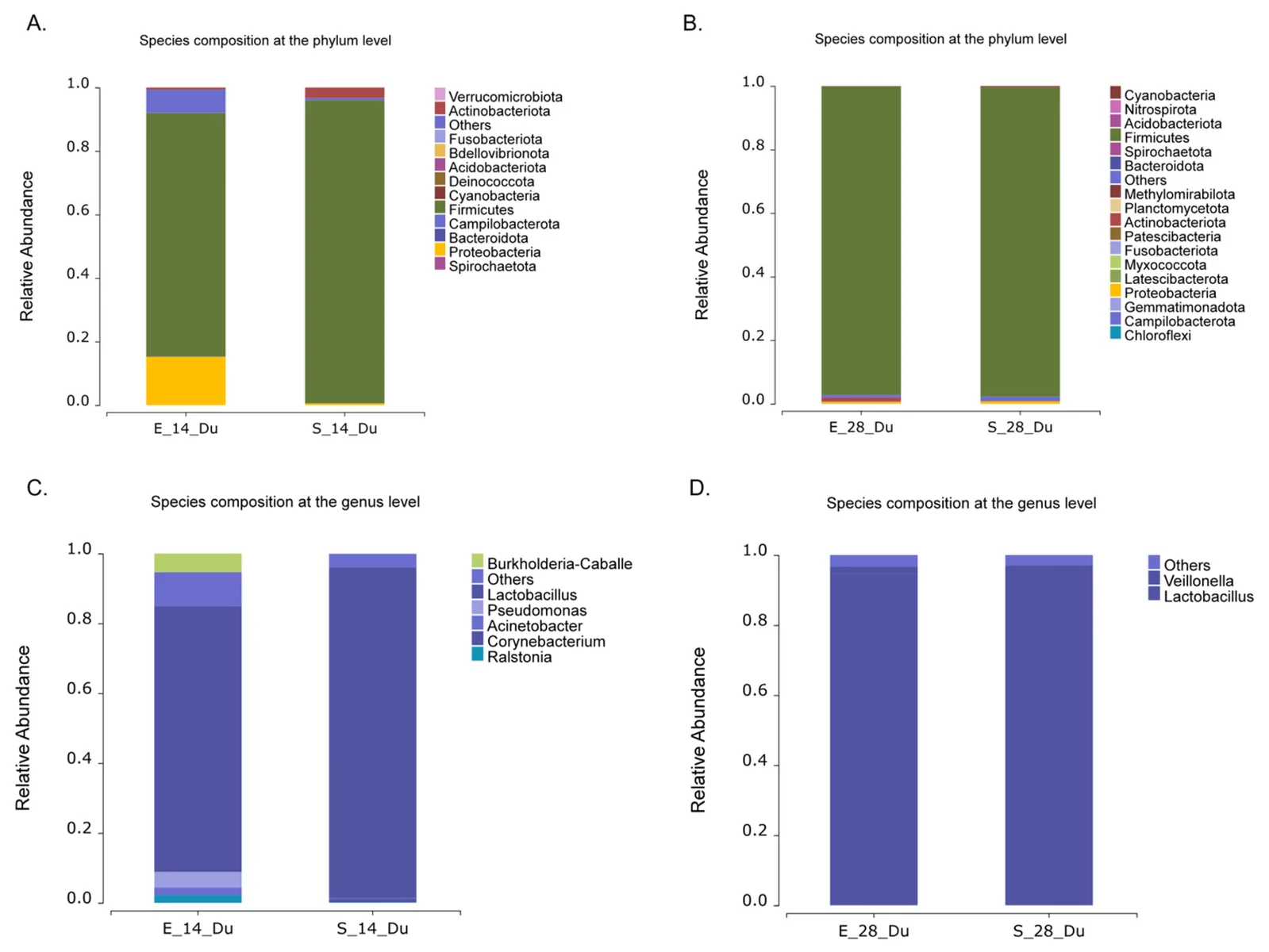
Stacked plots of relative abundance in the duodenal microbial community. (**A**) Relative abundance of taxa at the phylum level at 14 days of age. (**B**) Relative abundance of taxa at the phylum level at 28 days of age. (**C**) Relative abundance of taxa at the genus level at 14 days of age. (**D**) Relative abundance of taxa at the genus level at 28 days of age.

**Figure 7:**
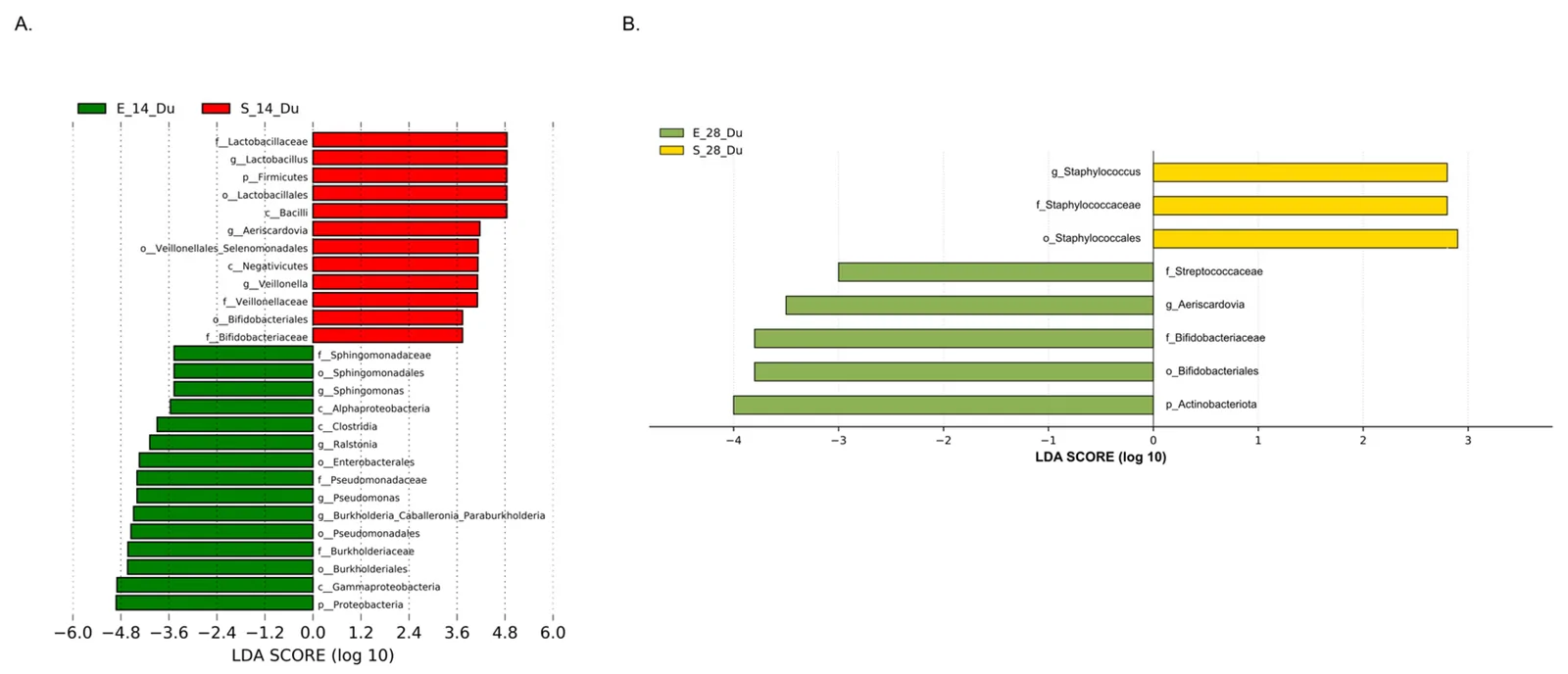
Bar plots of LDA scores for differentially abundant duodenal taxa identified by LEfSe in European meat pigeons and Shiqi pigeons at different ages. (**A**) Differential taxa between breeds at 14 days of age. (**B**) Differential taxa between breeds at 28 days of age. Green and light-green bars represented differential taxa enriched in European meat pigeons, whereas red and yellow bars represented differential taxa enriched in Shiqi pigeons. The LDA score (log10) was the log10-transformed LDA score. A higher absolute score indicated a more pronounced enrichment of the taxon in the corresponding group.

**Figure 8:**
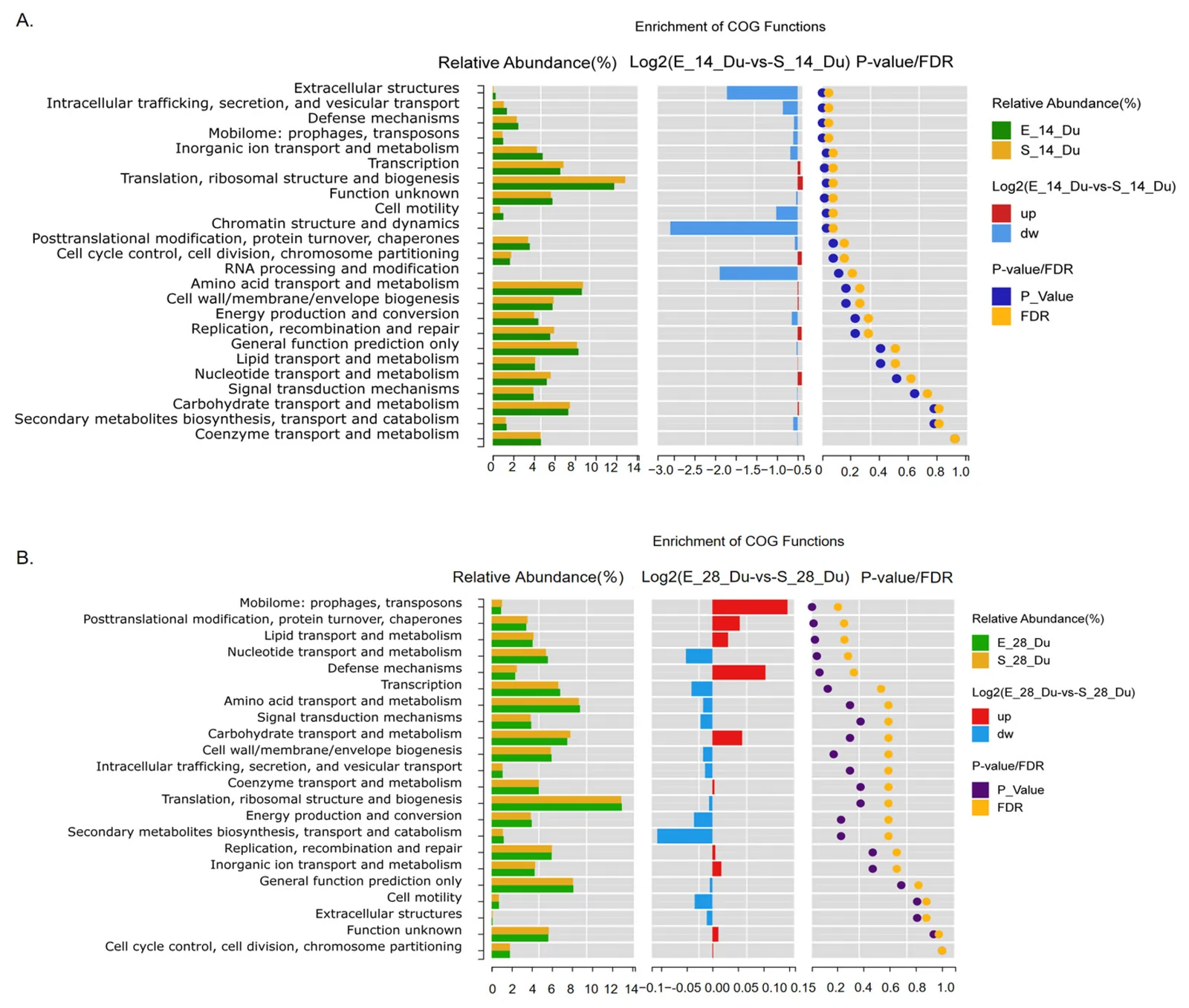
Three-panel plots showing differences in COG functional annotations of the duodenal microbiota between European meat pigeons and Shiqi pigeons at different ages. (**A**) COG analysis of the duodenal microbiota at 14 days of age. (**B**) COG analysis at 28 days of age. From left to right, each plot presents a bar chart of the relative abundance of COG functional categories, a bar chart of the log2 fold change between groups, and a *P*/FDR significance scatter plot. Green indicated functional abundance in European meat pigeons, and yellow indicated functional abundance in Shiqi pigeons. Red and blue bars indicated positive and negative log2 fold changes, respectively. Breed-specific abundance patterns are described from the relative-abundance bars in the left panel. Purple dots represented unadjusted *p* values, and yellow dots represented FDR-adjusted *p* values (*q* values). Functional profiles were predicted using PICRUSt2 from 16S rRNA gene sequences and represent inferred metabolic potential, not directly measured gene expression or metabolic activity.

**Figure 9:**
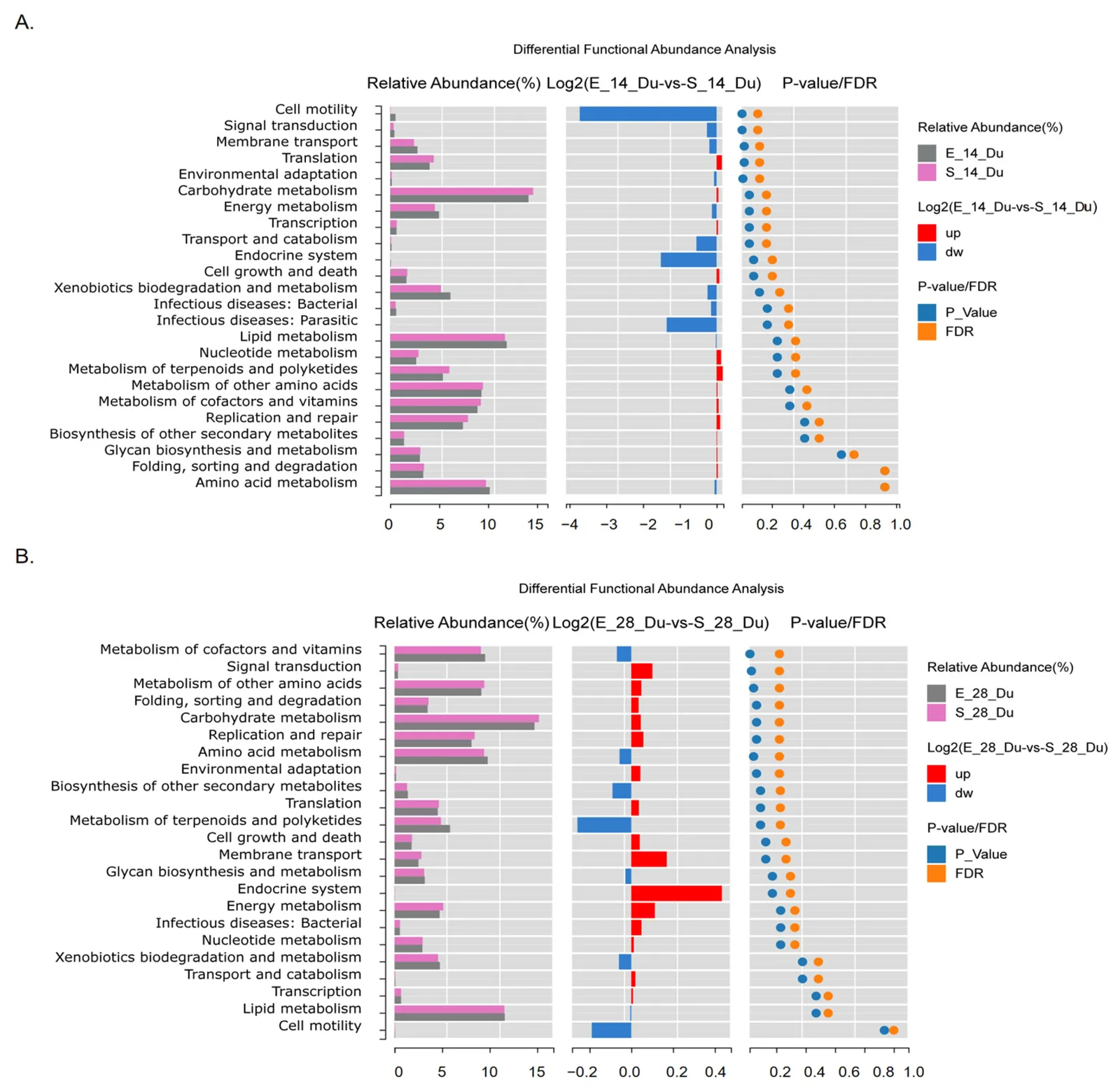
Three-panel plots showing differences in the abundance of level 2 KEGG pathways in the duodenal microbiota of European meat pigeons and Shiqi pigeons at different ages. (**A**) KEGG analysis at 14 days of age. (**B**) KEGG analysis at 28 days of age. From left to right, each plot presents a bar chart of relative pathway abundance, a bar chart of the log2 fold change between groups, and a *P*/FDR significance scatter plot. Gray indicated pathway abundance in European meat pigeons, and pink indicated pathway abundance in Shiqi pigeons. Red and blue bars indicated positive and negative log2 fold changes, respectively. Breed-specific abundance patterns are described from the relative-abundance bars in the left panel. Purple dots represented unadjusted *p* values, and yellow dots represented FDR-adjusted *p* values (*q* values). Functional profiles were predicted using PICRUSt2 from 16S rRNA gene sequences and represent inferred metabolic potential, not directly measured gene expression or metabolic activity.

**Figure 10:**
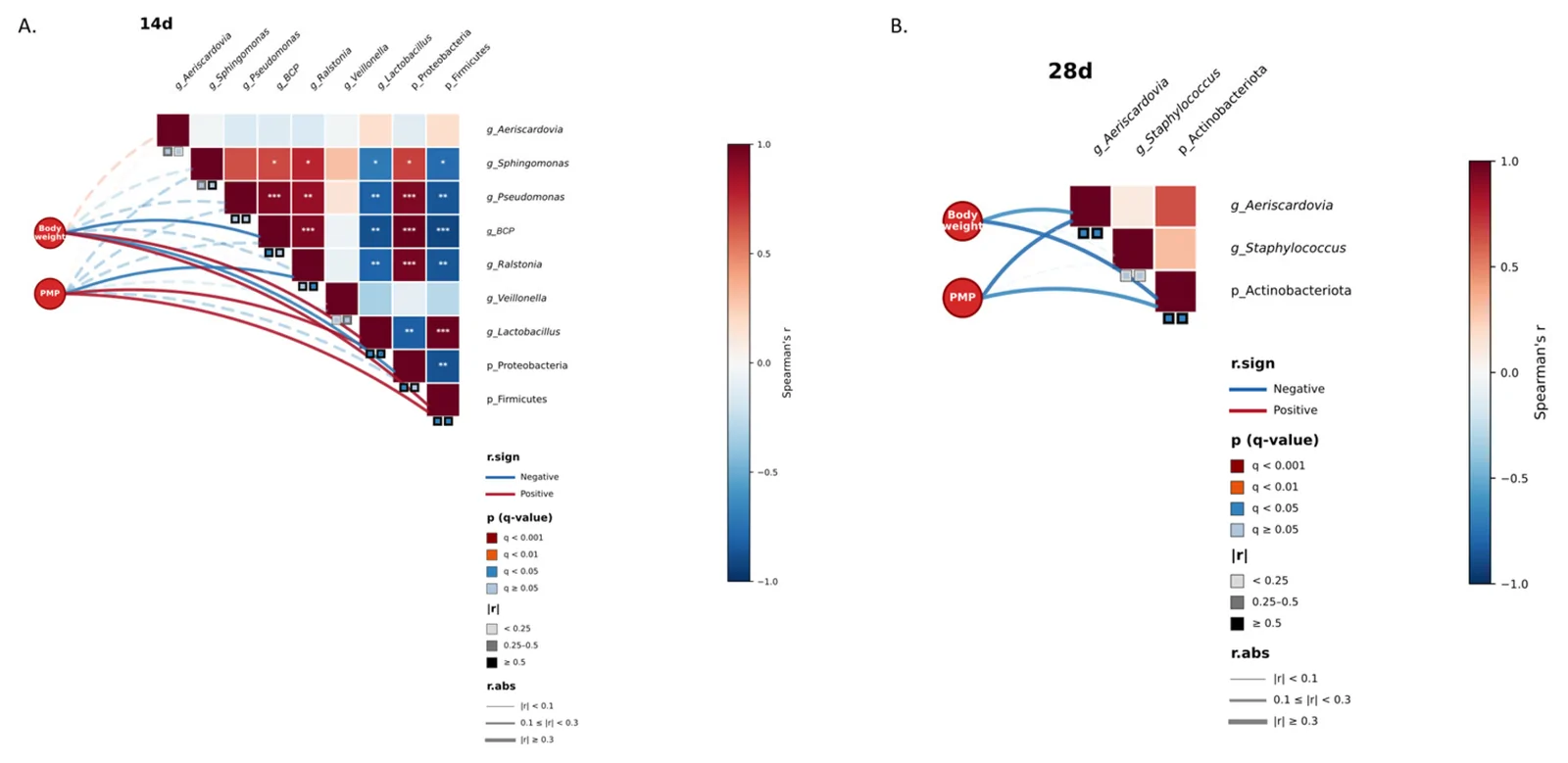
Spearman correlation matrices of differential taxa with live body weight (BW) and pectoral muscle percentage (PMP) in squabs. (**A**) Spearman correlations of differential taxa at the phylum and genus levels with growth traits at 14 days of age. (**B**) Spearman correlations at 28 days of age. BW, body weight; PMP, pectoral muscle percentage. g_BCP stands for g_Burkholderia-Caballeronia-Paraburkholderia. Red indicated a positive correlation, blue indicated a negative correlation, and darker colors indicated stronger correlations. Solid and dashed lines indicated significant and nonsignificant associations, respectively, based on the FDR threshold. * *q* < 0.05, ** *q* < 0.01, *** *q* < 0.001.

**Figure 11:**
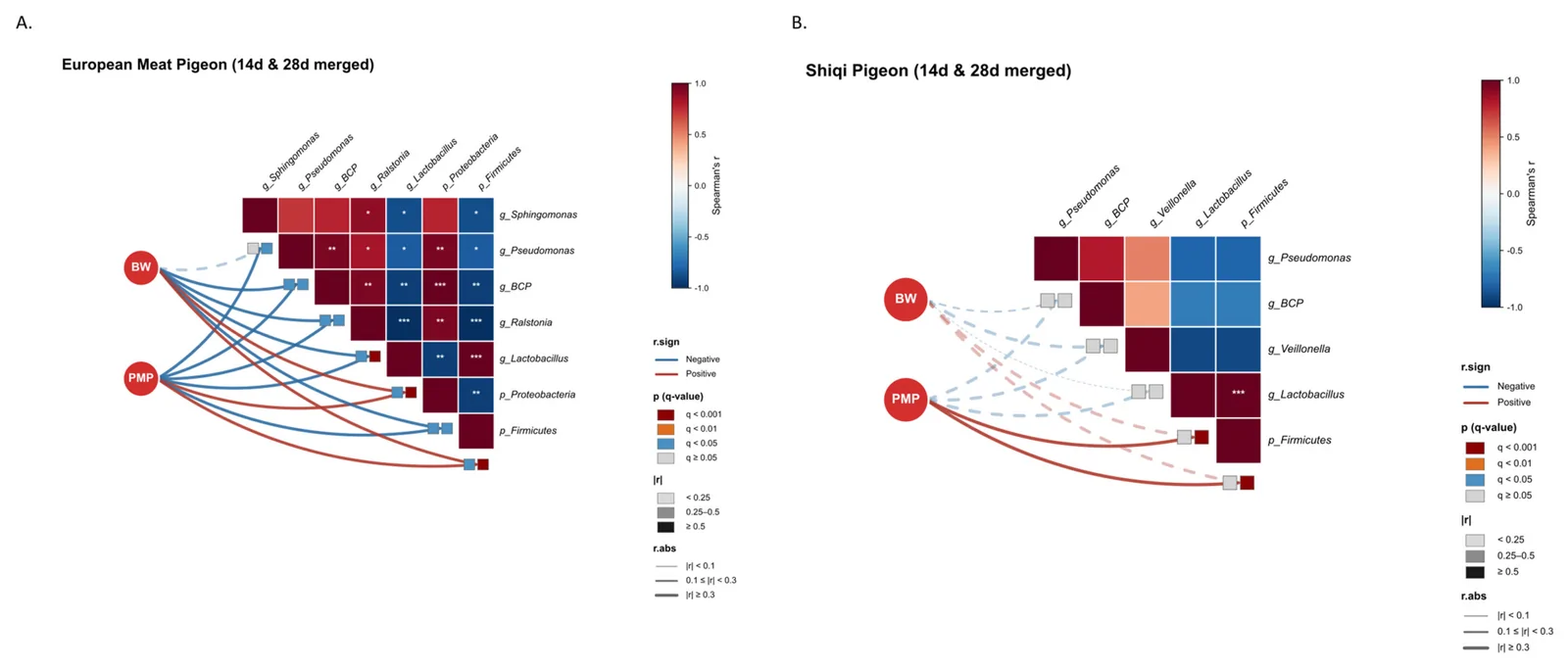
Spearman correlation matrices of differential taxa with live body weight (BW) and pectoral muscle percentage (PMP) in squabs. (**A**) Spearman correlations of differential taxa at the phylum and genus levels with growth traits in European meat pigeons. (**B**) Spearman correlations of differential taxa at the phylum and genus levels with growth traits in Shiqi pigeons. g_BCP stands for g_Burkholderia-Caballeronia-Paraburkholderia. Red indicated a positive correlation, blue indicated a negative correlation, and darker colors indicated stronger correlations. Solid and dashed lines indicated significant and nonsignificant associations, respectively, based on the FDR threshold. * *q* < 0.05, ** *q* < 0.01, *** *q* < 0.001.

## Data Availability

The data supporting this study are available in the GSA database under BioProject accession number PRJCA069765.

## Supplementary Materials

The following supporting information can be downloaded at: https://www.mdpi.com/article/10.3390/ani16193024/s1, Figure S1: Comparison of the Relative Abundance of the Top 10 Differential Taxa at the Phylum and Genus Levels in the Duodenal Microbiota of European Meat Pigeons and Shiqi Pigeons at Different Ages; Table S1: Effect sizes and confidence intervals for growth performance; Table S2: Data Filtering Table; Table S3: Relative Abundance of Taxa.
